# Celsior® vs. St Thomas® cardioplegia: analysis of myocardial protection and clinical safety in neonates

**DOI:** 10.3389/fped.2024.1430832

**Published:** 2024-07-08

**Authors:** Nicolae Cristian Bulescu, Julia Mitchell, Olivier Metton, Naoual El Jonhy, Camille Amaz, Thomas Perouse de Montclos, Marc Lilot, Nathan Mewton, Roland Henaine

**Affiliations:** ^1^ Congenital Cardiac Surgery, Louis Pradel Hospital, Lyon, France; ^2^ Center for Clinical Investigation, Louis Pradel Hospital, Lyon, France; ^3^ Pediatric and Congenital Cardiology Department, Louis Pradel Hospital, Lyon, France; ^4^ Pediatric Cardiac, Thoracic and Vascular Anesthesia and Intensive Care Unit, Louis Pradel Hospital, Lyon, France

**Keywords:** crystalloid cardioplegia, arterial switch operation, pediatric cardiac surgery, transposition of the great arteries (TGA), troponin

## Abstract

**Objective:**

To compare the effectiveness and safety of Celsior® crystalloid solution to St Thomas® solution as cardioplegia in pediatric arterial switch surgery.

**Methods:**

A retrospective study was conducted on 180 patients who underwent arterial switch operation (ASO) between 2005 and 2019. The patients were divided into two groups: the St Thomas group receiving St Thomas solution and the Celsior® group receiving Celsior® solution. The study aimed to assess myocardial protection while evaluating clinical outcomes of patients between groups.

**Results:**

Baseline characteristics not different between groups. The postoperative troponin release trends and blood lactate levels were not different between groups. However, the Celsior® group had a significant lower incidence of delayed sternal closure (9.7% vs. 19.5%; *p* = 0.09) and mechanical circulatory support (ECMO) (4.9% vs. 24.7%; *p* < 0.001) compared to the St Thomas group. The length of stay in the intensive care unit (ICU) was significantly shorter in the Celsior® group (4.6 ± 3.36 days vs. 8.72 ± 5.08 days, respectively; *p* < 0.001). There was no significant difference in 30-day mortality between the two groups (2.9% vs. 2.6%; *p* = 0.147).

**Conclusion:**

The study suggests that Celsior® solution is effective and safe for myocardial protection in pediatric arterial switch surgery. It may offer potential benefits such as reduced need for delayed sternal closure and ECMO support, as well as shorter ICU stay. However, the study has limitations including its retrospective design and the use of different cardioplegic solutions during different time periods. Further prospective randomized trials are needed for confirmation.

**Clinical Registration Number:**

ClinicalTrials.gov, ID: NCT04616222.

## Introduction

Adequate myocardial protection is one of the most essential elements for successful postoperative evolution in complex congenital heart surgery. The debate on the optimal solution between hyperkalemic blood formulations or crystalloid solutions is still open. Crystalloid solutions are an adapted resource for complex operations, as they have a longer duration of action and can be administered in a single dose without further interruptions.

Celsior® is a crystalloid extracellular-type solution. It has demonstrated its effectiveness in protecting the myocardium of transplanted hearts, where it was used as a preservation solution (1, 2). Recent studies have shown that Celsior® can be safely used in simple and complex adult cardiac surgery (3, 4).

Regarding the pediatric population, up to this point, there is no published data regarding the efficacy and the safety of Celsior® as a cardioplegic solution.

As Celsior® does not have official approval for use as a cardioplegic solution in the pediatric population in France, there is a need for clinical data regarding the safety and efficacy of myocardial protection in this patient population.

Our study had two principal objectives: to assess the myocardial protection with Celsior® compared to the traditional cardioprotective solutions and to evaluate its safety on clinical outcomes in a pediatric population undergoing arterial switch surgery.

### Materials and methods

This observational monocentric retrospective clinical study was conducted in the Cardiovascular Louis Pradel Hospital, a tertiary referral institution. The Hospices Civils de Lyon's clinical research ethics committee approved this study's implementation (institutional review board number 20–91). Informed consent was waived, but non-opposition to clinical data analysis was obtained from all participants in the current research. All variables were recorded in a computerized database.

### Study population

We started using Celsior® for the arterial switch operation (ASO) in 2012 at our institution. At that time, the clinical decision in favor of this cardioplegia solution was made mainly for complex operations requiring prolonged aortic cross-clamping times.

We included all consecutive patients who had an isolated arterial switch operation as a corrective procedure for transposition of the great arteries (TGA) from 2005 to 2019, using either cold Saint Thomas solution (the St Thomas group—from 2005 to 2011) or Celsior® cardioplegia (the Celsior® group—from 2012 to 2019). We excluded patients who had other significant cardiovascular anomalies that required surgical treatment at the same time as the ASO (such as ventricular septal defects or coarctation of the aorta); we also excluded patients with significant anomalies of the origin or course of the coronary arteries (single ostium—Yacoub type B or intramural course).

The procedures have been performed by the same consistent team of 3 pediatric cardiac surgeons.

### Myocardial protection protocol using the Saint Thomas solution

Two hundred milligrams of Procaine are added to the 1-L bag of St Thomas solution before use. After the aorta is cross-clamped, the cardioplegic solution is administered in the aortic root at a temperature of 4°C, at a total dose of 15 ml/kg. Once asystole is achieved, the right atrium is opened, and the cardioplegic solution is aspirated from the coronary sinus ostium.

A second dose and third dose of cardioplegia (at half-dose of the initial administration) are administered directly either into the coronary arteries, or the neo-aortic root afterreimplantation of the coronary arteries and reconnection of the neo-aortic root to the ascending aorta.

### Myocardial protection protocol using Celsior® cardioplegic solution

After the aorta is cross-clamped, Celsior® is administered in the aortic root at a temperature of 4°C, at a total dose of 15 ml/kg. Once asystole is achieved, the right atrium is opened, and the cardioplegic solution is aspirated from the coronary sinus ostium.

A second dose of cardioplegia (at half of the initial amount) is administered after finishing the “aortic” part of the repair.

### Surgical technique

All procedures were performed under general anesthesia and median sternotomy with aortic and bicaval cannulation. Reconstituted whole blood was used for priming (red blood cells + fresh frozen plasma); CPB was conducted under moderate hypothermia at 28°C core temperature. Target flow on CPB was 150 ml/kg at 36°C, and adjusted upon a multiparametric evaluation (temperature, mean arterial blood pressure, SvO_2_, Near InfraRed Spectroscopy values).

Upon institution of CPB, the ductus arteriosus was sutured and divided. The aorta was cross-clamped, and antegrade cardioplegia was instilled in the aortic root. Following right atriotomy, the cardioplegic effluent was aspirated from the coronary situs.

The ascending aorta was transected just above the sino-tubular junction and the coronary buttons are excised. The pulmonary artery is transected at the level of the bifurcation; the Lecompte maneuver is then performed.

The coronary arteries are reimplanted in the neoartic root and the continuity with the ascending aorta is reconstructed. At this point, a half-dose of cardioplegia is administered.

The atrial septal defect is then closed and the neopulmonary root is reconstructed with a patch of fresh, autologous pericardium. The aortic cross-clamp is removed, and the anastomosis with the pulmonary bifurcation is performed on a beating heart.

### Study endpoints

We assessed the effect of Celsior® cardioplegia compared to Saint Thomas on several parameters or clinical indices related to myocardial injury. These endpoints, surrogates of myocardial protection, were:

- Maximum postoperative serum troponin release, measured in the postoperative period at H4, H8, H24 after the return of the patient in the Intensive Care Unit (ICU)
- Maximal arterial blood lactate levels, measured in the postoperative period at H4, H8, H24 after the return of the patient in the ICU
- Need for mechanical circulatory support (ECMO) or delayed sternal closure (DSC),
- Postoperative rhythm disturbances, including atrial fibrillation, junctional rhythm, paroxysmal supraventricular tachycardia (PSVT), or atrioventricular block requiring external pacing,
- Length of stay in the ICU
- 30-day mortality.

We also compared the post-operative safety of Celsior® to St. Thomas on the following clinical endpoints:

- Bleeding: need for postoperative transfusion of red blood cells or surgical reexploration
- Renal toxicity—acute renal failure, defined as a postoperative increase of 50% in the creatinine level, compared to preoperative baseline levels.

Following a change in the hospital laboratory's biochemical technique, there was a shift from the assessment of Troponin I (TnI—ELISA, AA Architect Abbott) to ultra-sensitive Troponin I (TnI US—CMIA, Architect i2000 Abbott). This shift corresponded to the start of using the Celsior® solution in 2012. Consequently, in the St Thomas group, TnI (µg/L) was measured; in the Celsior® group, TnI US (ng/L) was measured. Because of this, the comparison in troponin levels between the Celsior® and Saint Thomas groups cannot be performed directly and is only indicative.

Data acquisition and analysis were performed with the help of the Center of Clinical Investigation. Electronic and hard-copy recordings have been used to retrieve the necessary data.

### Statistical analysis

Continuous variables are expressed as mean and standard deviations (SD) or as the median and minimum-maximum values; categorical variables are expressed as frequency. Student *t*-test and Chi-squared test (or Fischer exact test) were used, respectively, according to the type of data and the normal distribution for continuous data.

The surrogates of myocardial protection were assessed: maximum lactate and troponin release (in the first postoperative 24 h), delayed sternal closure and mechanical (ECMO) support, cardiac rhythm and onset of new arrhythmias, and 30-day mortality. A *p*-value < 0.05 was considered significant. All statistical analyses were performed using Jasp R based open-source interface (0.17.3, https://jasp-stats.org, University of Amsterdam, Netherlands).

## Results

### Study population

The study group included 180 patients operated between 2005 and 2019 for complete anatomical correction of isolated D-transposition of the great arteries (D-TGA). St Thomas was the cardioplegic solution used between 2005 and 2011 in 77 patients (the St Thomas group). Celsior® was used in 103 patients, between 2012 and 2019 (Table 1).

**Table 1:** Preoperative and operative characteristics; CPB: cardio-pulmonary by-pass.

	Overall (*n* = 180)	St Thomas (*n* = 77)	Celsior® (*n* = 103)	*p* value
Preoperative characteristics				
Age (days), mean ± SD	6.3 ± 3.2	5.9 ± 3.6	6.7 ± 2.8	0.007
Male sex, *n* (%)	126 (70%)	53 (68.8%)	73 (70.9%)	0.870
Weight (kg), mean ± SD	3.24 ± 0.5	3.1 ± 0.5	3.2 ± 0.5	0.100
Preoperative Rashkind, *n* (%)	153 (85%)	68 (88.3%)	85 (82.5%)	0.054
Preoperative intubation, *n* (%)	56 (31.1%)	37 (48.1%)	19 (18.4%)	<0.001
Altered LV function, *n* (%)	49 (27.2%)	24 (31.2%)	25 (24.3%)	0.103
Preoperative creatinine levels (µmol/L), mean ± SD	58.4 ± 24.9	64.5 ± 18.2	53.8 ± 28.2	<0.001
Operative characteristics				
CPB time (minutes), mean ± SD	119.5 ± 33.8	114.9 ± 21.2	122.9 ± 40.5	0.272
Cross-clamp time (minutes), mean ± SD	77.1 ± 19.5	74.6 ± 9.9	78.9 ± 24.2	0.189
Minimum core temperature (^o^C), mean ± SD	29.8 ± 2.9	29.1 ± 2.1	30.4 ± 3.3	0.026

There were no statistically significant differences in sex, age (in days) or weight, or the use of a preoperative Rashkind maneuver between the St Thomas group and the Celsior® group. Patients in the St Thomas group had a greater incidence of preoperative intubation (*p* < 0.001) and higher preoperative creatinine levels (*p* < 0.001).

### Intraoperative results

CPB duration (114.8 ± 21.2 vs. 122.9 ± 40.5 min, *p* = 0.27) and aortic cross-clamp time (74.5 ± 9.9 vs. 78.2 ± 24.2 min, *p* = 0.18) were slightly shorter in the St Thomas group compared to the Celsior group, without statistical significance. The mean minimum temperature achieved during CPB was lower in the St Thomas group than in the Celsior group (*p* = 0.026).

The mean volume of administered cardioplegia was similar in the St Thomas and the Celsior groups (195.99 ± 85.97 ml vs. 175.28 ± 61.81 ml, respectively, *p* = 0.11).

### Myocardial protection endpoints

The maximal troponin levels between the St Thomas and Celsior® groups were not comparable because of the change in laboratory assays in 2012. The maximal level in the St Thomas group (TnI) was: 12,125 ± 9,636.8 µg/ml. In the Celsior® group (TnI US) the maximal troponin level was 13,293.4 ± 12,157.5 ng/ml.

**Figure 1:**
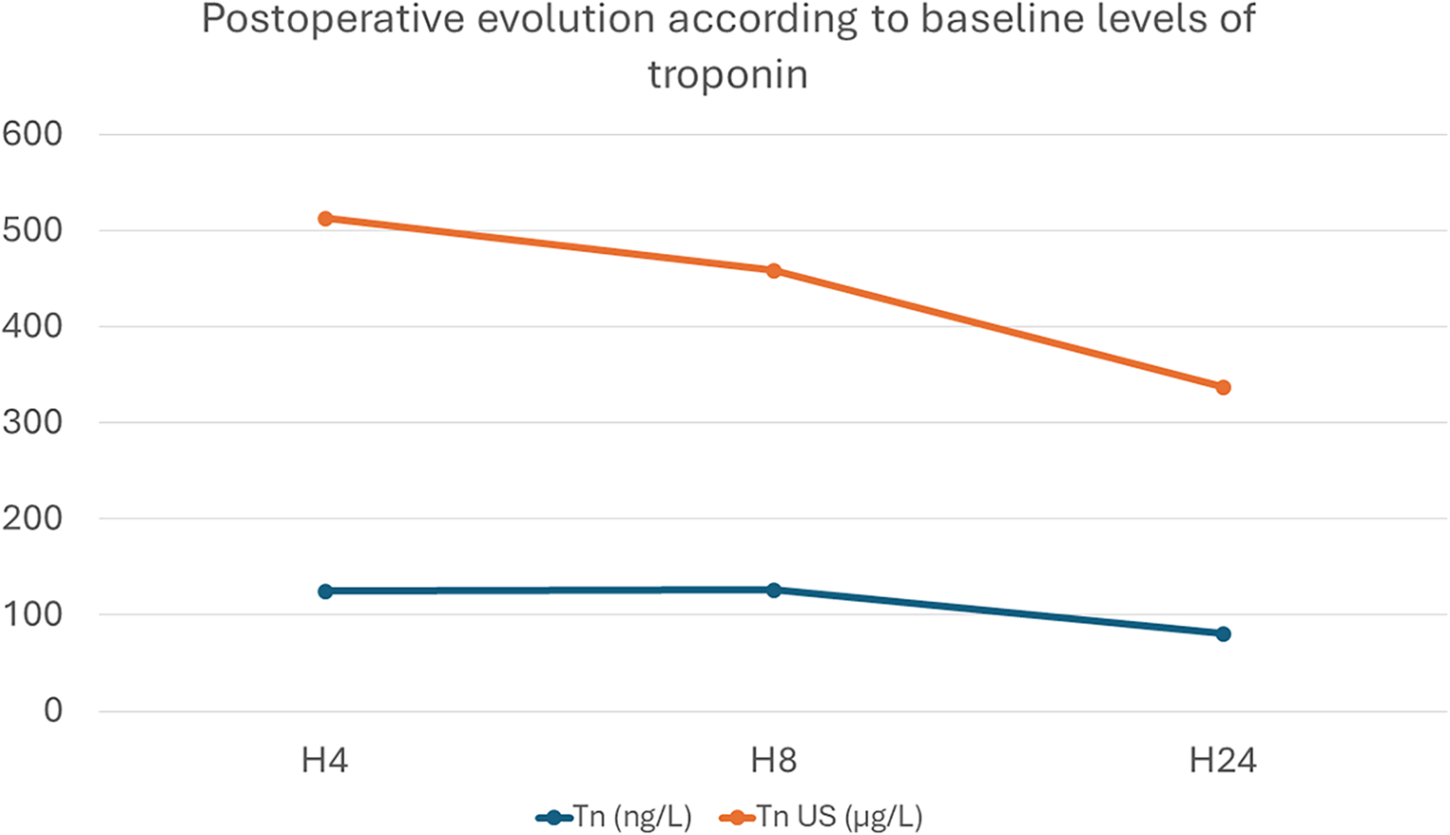
Evolution of troponin levels, expressed as number of times it has increased in relation to baseline levels: Tn (ng/L) was measured in the St Thomas group; Tn US (µg/L) was used in the Celsior® group.

As reported in Figure 1, we assessed the two groups by comparing the troponin level evolution in relation to the baseline values: 0.100 µg/L for TnI vs. 26 ng/L for TnI US. The temporal trend was similar between both groups, with a peak troponin release at H4 and a descending pattern by H24 (Figure 1).

**Figure 2:**
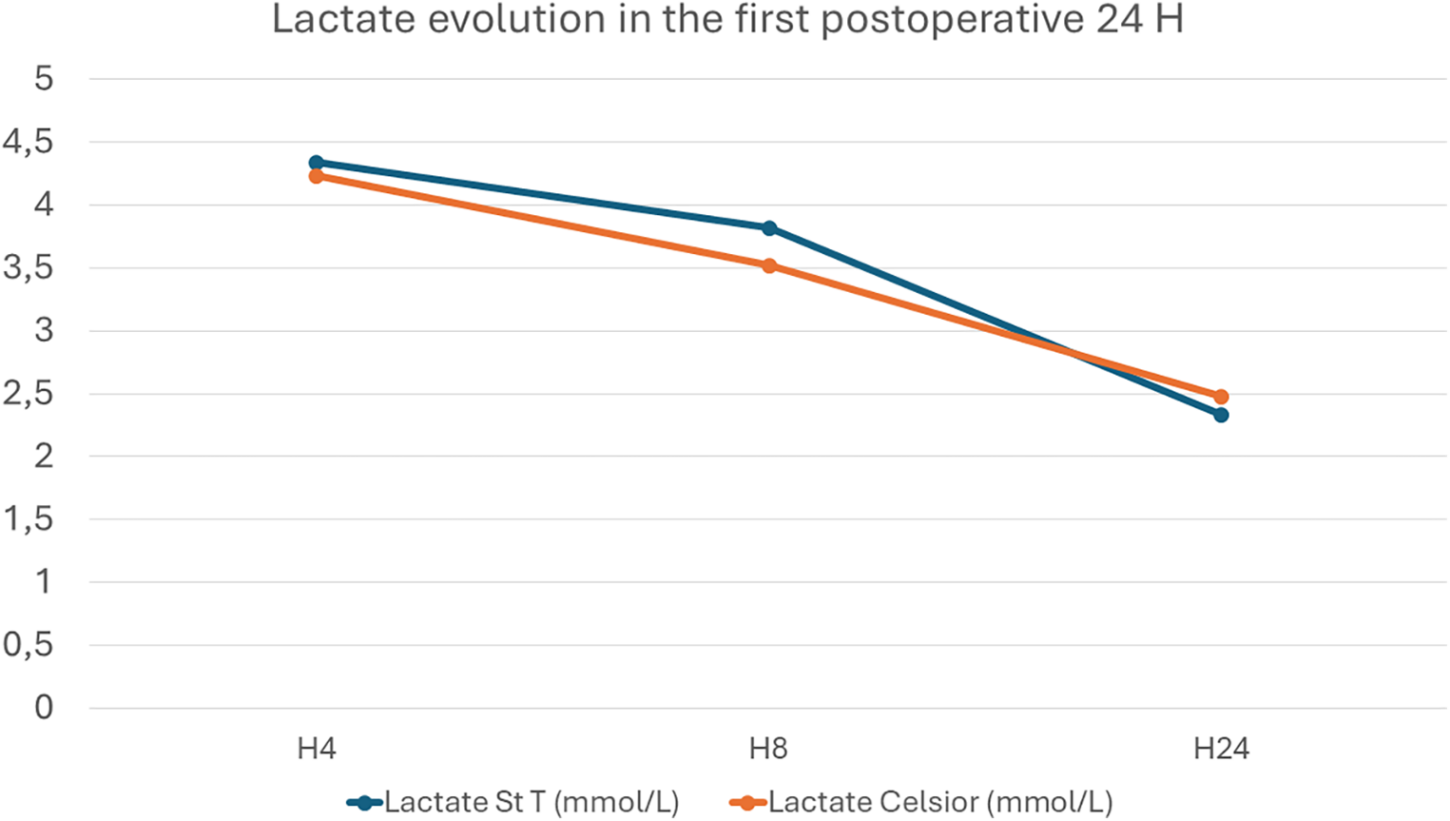
Evolution of mean lactate levels in the first 24 postoperative hours.

There was no significant difference in the maximum blood lactate level in the first POD between the St Thomas and the Celsior® groups (4.1 ± 2.1 vs. 4.26 ± 1.7; *p* = 0.29). The lactate curve had a similar descending pattern in both groups (Figure 2).

Fifteen patients in the St Thomas group (19.5%) and 10 patients in the Celsior® group (9.7%) required delayed sternal closure (*p* = 0.099). The chest was closed in the ICU after a median of 3.5 days in both groups (*p* = 0.779) (Table 2).

**Table 2:** Myocardial protection and safety of the 2 cardioplegic solutions.

	Overall (*n* = 180)	St Thomas (*n* = 77)	Celsior® (*n* = 103)	*p* value
Maximum lactate level, mmol/L	4.2 ± 1.8	4.1 ± 2.1	4.26 ± 1.7	0.288
Delayed sternal closure, *n* (%)	25 (13.9%)	15 (19.5%)	10 (9.7%)	0.099
ECMO use, *n* (%)	24 (13.3%)	19 (24.7%)	5 (4.9%)	<0.001
Arrhythmias	17 (9.4%)	9 (11.7%)	8 (7.8%)	0.304
Atrial fibrillation	4 (2.2%)	3 (3.9%)	1 (1%)	
Junctional rhythm	10 (5.6%)	3 (3.9%)	7 (6.8)	
PSVT	2 (1.1%)	2 (2.6%)	0 (0%)	
Other	0 (0%)	1 (1.3%)	0 (0%)	
ICU stay, mean (days)	6.3 ± 4.6	8.7 ± 5	4.6 ± 3.3	<0.001
Surgical reexploration for bleeding, *n* (%)	2 (1.1%)	1 (1.3%)	1 (1%)	0.331
Total transfusion volume, ml, mean + SD	106.1 ± 185.3	123.7 ± 250.5	87.2 ± 63.2	0.898
Creatinine H24 (µmol/L), mean + SD	66.3 ± 25.2	67.3 ± 18	65.5 ± 29.7	0.033
Absolute variation of creatinine (µmol/L), preop vs. H24, mean + SD	8.1 ± 19.9	2.4 ± 15.8	12.5 ± 21.7	0.003

The use of temporary mechanical circulatory support (ECMO) was required in 19 patients from the St Thomas group (24.7%) and 5 patients from the Celsior® group (4.9%). This difference was statistically significant (*p* < 0.001).

Arrhythmias occurred in 9 patients from the St Thomas group and in 8 patients from the Celsior® group (*p* = 0.304). Atrial fibrillation and junctional rhythm were the most frequent arrhythmias reported (Table 2).

The mean length of stay in the ICU was significantly longer in the St Thomas group than in the Celsior® group (8.72 ± 5.08 vs. 4.6 ± 3.36 days, respectively; *p* < 0.001).

### Mortality

The overall mortality in the study group was 2.77% (5 patients): two patients in the St Thomas group (2.6%) and three patients in the Celsior® group (2.9%) (*p* = 0.147).

### Safety of Celsior® solution

There were no allergic reactions encountered in the 2 groups.

Surgical reexploration for bleeding was required in only one patient from each group. The RBC transfusion volume was slightly higher in the St Thomas group compared to the Celsior group, without reaching statistical significance (123.7 ± 250.5 ml vs. 87.2 ± 63.2 ml, *p* = 0.898).

Regarding the renal function, there was a higher absolute increase of the postoperative creatinine level in the Celsior® group (12.59 ± 21.72 mmol/L) vs. the St Thomas group (2.45 ± 15.8 µmol/L) (*p* = 0.033), but it was below the 50% variation from the preoperative creatinine level (53.87 ± 28.24 µmol/L vs. 64.56 ± 18.23 µmol/L).

## Discussion

Effective myocardial protection via cardioplegia administration is of paramount importance in cardiac surgery. There are almost 70 years of experience using cardioplegic solutions, and new formulations are being introduced regularly. There is, however, no evidence of superiority of a specific solution compared to others, nor for an optimal temperature of administration (5, 6). Our results in a retrospective analysis of a pediatric patient population suggest no safety issue with using Celsior as a cardioplegic solution compared to St Thomas. Our study further suggests a potential benefit on post-operative outcomes, but this should be confirmed in a proper and adequately powered randomized controlled trial.

Historically, blood cardioplegia preparations have been the first to be used in cardiac surgery. Although they are effective and have a good safety margin, their short duration of action, of only 20–30 min, requires repeated administration, producing interruptions of the main surgical procedure. Conversely, an antegrade dose of Celsior® is effective for 40–60 min. This is especially important in the ASO, where the aortic part of the operation is normally finished well within this time frame. This allows for sufficient time for careful assessment of the origin and distribution of the coronary arteries, their detachment from the aortic root, and their subsequent correct reimplantation into the neo-aortic root; this step of the operation has been shown to be the most important predictive factor regarding short- and long-term mortality following the ASO (7, 8). It should be performed without interrupting it to administer a new dose of cardioplegia.

The Celsior® solution was introduced in 1994 for solid organ preservation (9) and is currently used for myocardial protection in adult and pediatric donor-harvested hearts (10). There are also reports of it being used for myocardial protection during conventional and minimally invasive cardiac surgery. Most of these reports focus on adults (3, 4, 11). There is a lack of knowledge regarding its' clinical effectiveness and safety in children, infants and neonates; published papers have focused on ultrastructural changes following cardioplegia administration (12) and its effectiveness as an organ preservation solution. To the best of our knowledge, no clinical studies have been conducted for the cardioplegic use in the pediatric population.

Postoperative troponin release is a sensitive and specific biomarker of myocardial injury and damage after cardiac surgery. There are postoperative variations of troponin values that have been well documented in different surgical procedures, but looking specifically to the ASO (13); in this study, using the St Thomas solution in simple TGA cases, the peak value is encountered 4 h (H + 4) after cross-clamp removal. Peak troponin is correlated with the duration of inotropic support, ventilation, and ICU stay. We found the same trends in postoperative release, with a peak at postoperative H + 4 followed by a marked decline by H + 8 and H + 24. The absolute variation of measured troponin was higher with the US TnI, as it is a more sensitive biomarker (14), but the same trend was observed in both types of troponins analyzed. The same trends in absolute postoperative increase and progressive decline have been observed in the adult population (4, 15). As the introduction of the US TnI assay corresponded roughly to the introduction of Celsior® in our day-to-day practice, we cannot affirm that the new solution had any significant impact on postoperative troponin release and no adverse effect on myocardial protection.

Further equivalence between St Thomas and Celsior® is suggested by the maximum blood lactate levels, which are similar in both groups. However, our study suggests the benefits of Celsior® in terms of the absence of low cardiac output, assessed by DSC and ECMO use. There is an almost twofold decrease of DSC incidence in the Celsior® group compared to the St Thomas group. Patients with a delayed sternal closure have an associated morbidity and surgical wound infection (16) and a mortality rate that is 3 times higher than patients who have a closed chest (17).

The need for temporary circulatory assistance is associated with a markedly increased mortality rate (18). In the setting of ASO, low cardiac output can have anatomical reasons (defective coronary reimplantation techniques, neo-aortic valve regurgitation, pulmonary artery branch stenosis due to the Lecompte maneuver) or physiological reasons (ischemia-reperfusion lesions or insufficient myocardial protection), which can be caused by an inadequate cardioplegic solution. In our study, there was a five-fold decrease in the need for postoperative ECMO in the Celsior® group, again suggesting better myocardial protection than with St Thomas.

To this end, there is arguably a contribution to the overall increase in performance of our surgical and medical team over the 15 years of enrollment; we believe, however, that the human factor was not the most important one, and the contribution of the Celsior® solution was of great importance into improving our results.

Blood transfusion requirement was higher in the St Thomas group, perhaps linked with the increased incidence of DSC and ECMO use, both situations where there is an increased postoperative hemorrhage risk. Postoperative bleeding requiring surgical reexploration was minimal in both groups.

There was a slight postoperative increase in serum creatinine levels, and the absolute variation was higher in the Celsior® group compared to the St Thomas group. However, this increase in creatinine was not clinically relevant. In addition, the baseline value was significantly higher in the St Thomas group, and the peak levels at H + 24 were equivalent between both groups. We, therefore, think that this significant increase does not have any clinical relevance and that this data does not support increased renal toxicity with Celsior®.

As there was no increased incidence of allergic, hematologic or renal adverse reaction in the Celsior® group, we can affirm that, in our observational study, there is no proof of maleficence associated with the use of this cardioplegic solution in the pediatric population.

## Study limitations

The main limitation of our study, other than its retrospective monocentric design, is that the two cardioplegic solutions were not used during the same time frame. Although it was the same surgical team that performed the procedure, the overall surgical, anesthesiologic, and intensive care performance has improved over the 15 years spanning our inclusion period. Although the surgical procedure has not seen any major change, there have been small adjuncts in these patients' intraoperative and postoperative care, and surely a learning and improvement curve, which may have partly been responsible for increased performance.

On the other hand, the fact that the troponin measurement technique changed during the study period means that we could not directly compare the same biochemical marker in our two study groups. We believe however that this is a valid tool for assessing the myocardial protection properties of both solutions. Further prospective clinical randomized trials should be performed to demonstrate and confirm this potential benefit with solid evidence.

## Conclusion

In a retrospective monocentric study, using the Celsior® cardioplegic solution seemed effective and safe for complex pediatric heart surgery procedures with comparable performance compared to the St Thomas crystalloid solution. Our neonatal cardiac surgery patient population had no observed toxicity or adverse events. On the contrary, our study suggested significant benefits in post-operative care. It has proved to be a strong alternative to blood cardioplegia or other widely used crystalloid cardioplegic solutions. Further prospective clinical randomized trials should be performed to confirm this benefit.

## Data Availability

The raw data supporting the conclusions of this article will be made available by the authors, without undue reservation.
